# Bone Protective Effect of Extra-Virgin Olive Oil Phenolic Compounds by Modulating Osteoblast Gene Expression

**DOI:** 10.3390/nu11081722

**Published:** 2019-07-25

**Authors:** Lucía Melguizo-Rodríguez, Francisco Javier Manzano-Moreno, Rebeca Illescas-Montes, Javier Ramos-Torrecillas, Elvira de Luna-Bertos, Concepción Ruiz, Olga García-Martínez

**Affiliations:** 1 Biomedical Group (BIO277), Department of Nursing, Faculty of Health Sciences, University of Granada, Avda. Ilustración 60, 18016 Granada, Spain; 2 Instituto Investigación Biosanitaria, ibs.Granada, C/ Doctor Azpitarte 4, 4ª planta, 18012 Granada, Spain; 3 Biomedical Group (BIO277), Department of Stomatology, School of Dentistry, University of Granada, Colegio Máximo, Campus Universitario de Cartuja, 18071 Granada, Spain; 4 Institute of Neuroscience, University of Granada, Centro de Investigación Biomédica (CIBM). Parque de Tecnológico de la Salud (PTS) Avda. del Conocimiento S/N, 18016 Armilla, Granada, Spain

**Keywords:** extra virgin olive oil, phenolic compounds, gene expression, osteoblast, genetic profile

## Abstract

The phenolic compounds of extra-virgin olive oil can act at various levels to protect individuals against cardiovascular and neurodegenerative diseases, cancer, and osteoporosis, among others. Polyphenols in extra-virgin olive oil can stimulate the proliferation of osteoblasts, modify their antigen profile, and promote alkaline phosphatase synthesis. The objective of this work was to determine the effect of different extra-virgin olive oil phenolic compounds on the gene expression of osteoblast-related markers. The cells of the MG63 osteoblast line were cultured for 24 h with 10^−6^ M of the phenolic compounds ferulic acid, caffeic acid, coumaric acid, apigenin, or luteolin. The expression of studied markers was quantified using quantitative real-time polymerase chain reaction (q-RT-PCR). The expression by MG63 osteoblasts of growth and differentiation/maturation markers was modified after 24 h of treatment with 10^−6^ M of the phenolic compounds under study, most of which increased the gene expression of the transforming growth factor β1 (TGF-β1), TGF-β receptor 1,2 and 3 (TGF-βR1, TGF-βR2, TGF-βR3), bone morphogenetic protein 2 and 7 (BMP2, BMP7), run-related transcription factor 2 (RUNX-2), Alkaline phosphatase (ALP), Osteocalcin (OSC), Osterix (OSX), Collagen type I (Col-I) and osteoprotegerin (OPN). The extra-virgin olive oil phenolic compounds may have a beneficial effect on bone by modulating osteoblast physiology, which would support their protective effect against bone pathologies.

## 1. Introduction

The Mediterranean diet is a balanced and varied nutritional model characterized by the consumption of fruit, vegetables, polyunsaturated fats (e.g., from fish), and monounsaturated fats (e.g., from dried fruit, nuts, and olive oil) [1]. Extra-virgin olive oil (EVOO), a key component of this diet, contains more than 30 phenolic compounds with antioxidant capacities [2,3]. The chemical composition of EVOO comprises a majority fraction, mainly triglycerides, and a minority fraction that includes phenolic compounds [4]. EVOO appears to meet the definition of a functional food proposed by the International Life Sciences Institute (ILSI Europe) in 1993, given its nutritional properties and its beneficial effect on one at least one function of the organism [5]. It has been reported that the phenolic compounds in EVOO can protect individuals against cardiovascular and neurodegenerative diseases, cancer, and osteoporosis, among others [6,7,8,9].

Osteoporosis is characterized by bone mass loss and bone microarchitecture impairment [10] and affects approximately 200 million women worldwide [11], causing more than 8.9 million fractures a year [12]. It results from an imbalance between bone formation and resorption mechanisms [13,14], with the osteoblast playing a key role as the main cell promoting bone tissue formation and repair. Osteoblasts derive from mesenchymal cells, and their physiology and activity are widely influenced by endocrine, autocrine, and paracrine factors [15].

Phenolic compounds in olive oil, such us apigenin, luteolin, coumaric acid, ferulic acid and caffeic acid, have been found to increase the proliferative capacity and differentiation of osteoblasts, among other effects, being 10^−6^ M of the most active dose between the studied concentrations [6,16,17]. However, the underlying mechanisms of their action remain unknown. Studies of markers related to bone resorption (e.g., osteoprotegerin [OPG] and OPG/receptor activator of nuclear factor-kappaB ligand [RANKL]) have demonstrated that phenolic compounds in EVOO inhibited osteoclastogenesis, and therefore bone tissue loss [18,19]. It has also been observed that the EVOO phenolic compound oleuropein favored bone preservation and repair by increasing the expression of genes related to osteoblastogenesis, including RUN-related transcription factor 2 (RUNX-2), osterix (OSX), collagen type I (COL-I), osteocalcin (OSC), and alkaline phosphatase (ALP) [20], thereby favoring bone preservation and repair.

The aim of this study was to explore the action mechanisms that underlie the protective effects on bone tissue of EVOO phenolic compounds by studying the gene expression of osteoblast-related markers, including bone morphogenetic proteins 2 and 7 (BMP-2 and BMP-7), transforming growth factor (TGF)-β1, TGF-β receptors (TGF-βR1, TGF-βR2; TGF-βR3), RUNX-2, ALP, COL-I, OSX, OPG and OSC, using the MG63 osteoblastic cell line.

## 2. Material and Methods

### 2.1. Chemical Compounds

Caffeic acid, ferulic acid, coumaric acid, luteolin and apigenin were purchased from Sigma-Aldrich (St. Louis, MO, USA) and kept at −20 °C. The aliquots of these phenolic compounds were prepared from mother solutions to achieve the appropriate concentration for each treatment. All solvents used were of analytical or HPLC grade (Sigma-Aldrich), and the water was of Milli-Q quality (Millipore Corp, Bedford, MA, USA).

### 2.2. Cell Culture

The MG63 osteoblast cell line, derived from a 14-year-old Caucasian male, was obtained from the ATCC. The cells were preserved in Dulbecco’s Modified Eagle Medium (DMEM; Invitrogen Gibco Cell Culture Products, Carlsbad, CA, USA) with 100 IU/mL penicillin (Lab Roger SA, Barcelona, Spain), 50 μg/mL gentamicin (Braun Medical SA, Jaen, Spain), 2.5 μg/mL amphotericin B (Sigma, St Louis, MO, USA), 1% glutamine (Sigma), and 2% HEPES (Sigma) supplemented with 10% fetal bovine serum (FBS) (Gibco, Paisley, UK). The cultures were kept at 37 °C in a humidified atmosphere of 95% air and 5% CO2. The cells were separated from the culture flask using 5 mL of a solution of 0.05% Trypsin (Sigma) and 0.02% ethylenediaminetetraacetic acid (EDTA) (Sigma) followed by their neutralization and re-suspension in a complete culture medium with 10% FBS.

### 2.3. Treatments

The osteoblasts were seeded at 1 × 10^4^ cells/ml per well into a 24-well plate (Falcom, Becton Dickinson Labware, Franklin Lakes, NJ, USA) and cultured at 37 °C in a humidified atmosphere of 95% air and 5% CO_2_ until a 80% of confluence of the well was reached. Then, the media was replaced with DMEM containing phenolic compounds treatment at 10^−6^ M. All experiments included cells incubated under the same conditions without treatment compounds as an internal control. Three separate experiments were performed for each treatment and at least every experiment was performed in triplicate.

### 2.4. RNA Extraction and cDNA Synthesis (Reverse Transcription)

The mRNA was extracted from the cells by a silicate gel technique using the Qiagen RNeasy extraction kit (Qiagen Inc., Hilden, Germany), which includes a DNAse digestion step. The amount of extracted mRNA was measured by UV spectrophotometry at 260 nm (Eppendorf AG, Hamburg, Germany), and contamination with proteins was determined according to the 260/280 ratio. An equal amount of RNA (1 μg total RNA in 40 μL total volume) was reverse-transcribed to cDNA and amplified by PCR with the iScript™ cDNA Synthesis Kit (Bio-Rad laboratories, Hercules, CA, USA), following the manufacturer’s instructions.

### 2.5. Real-Time Polymerase Chain Reaction (RT-PCR)

The mRNA of RUNX-2, OSX, OSC, COL-I, BMP-2, BMP-7, TGFβ1, TGFβ-R1, TGFβ-R2 and TGFβ-R3 was detected with primers designed using the NCBI-nucleotide library and Primer3-design (Table 1). All primers had been matched to the mRNA sequences of the target genes (NCBI Blast software). The final results were normalized using ubiquitin C (UBC), peptidylprolyl isomerase A (PPIA), and ribosomal protein S13 (RPS13) as stable housekeeping genes [21].

### 2.6. Statistical Analysis

Further, SPSS 22.0 (IBM, Chicago, IL, USA) was used for statistical analyses. The mRNA levels were expressed as the means ± standard deviation (SD). The multiple analysis of variance was applied for data comparisons, considering *p* < 0.05 to be significant. All studies were conducted in triplicate.

## 3. Results

### 3.1. Effect of Phenolic Compounds on Gene Expression of TGFβ1 and its Receptors (TGFβ- R1, TGFβ-R2, and TGFβ-R3)

**Figure 1:**
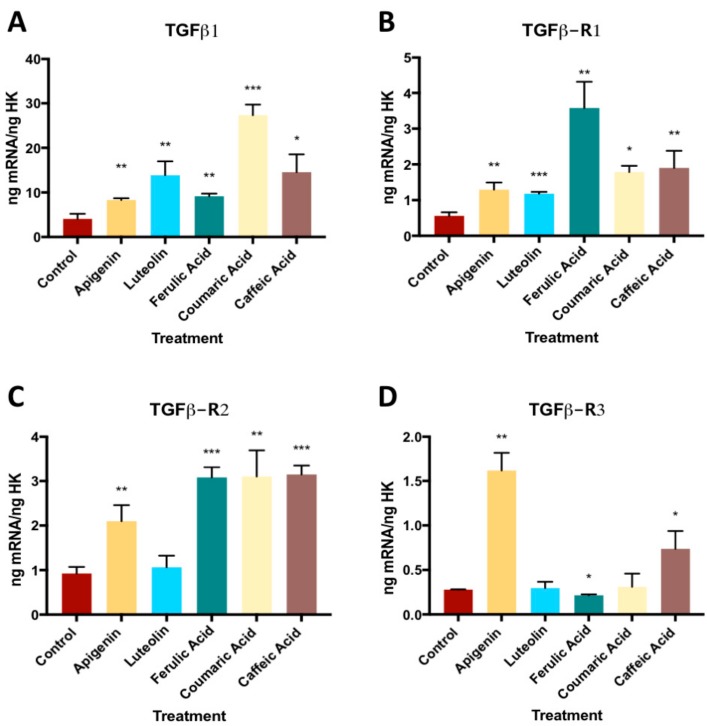
The effect of phenolic compounds on gene expression of TGFβ1 and its receptors (TGFβ-R1, TGFβ-R2, and TGFβ-R3). * *p* < 0.05, ** *p* < 0.005, *** *p* < 0.001. TGF-β1: Transforming growth factor β1, TGF-β receptor 1,2 and 3 (TGF-βR1, TGF-βR2, TGF-βR3).

The treatment of the MG63 osteoblast line with 10^−6^ M caffeic acid, ferulic acid, coumaric acid, apigenin, or luteolin for 24 h significantly increased the expression of TGFβ1 and its receptor TGFβ-R1, as observed in Figure 1. The expression of receptor TGFβ-R2 significantly increased by the treatment with all phenolic compounds, except for luteolin, which had no significant effect with respect to untreated control cells. The expression of receptor TGFβ-R3 was significantly increased by the treatment with all phenolic compounds, except for luteolin and coumaric acid (See Supplementary Table S1).

### 3.2. Effect of Phenolic Compounds on Gene Expression on BMP2 and BMP7

**Figure 2:**
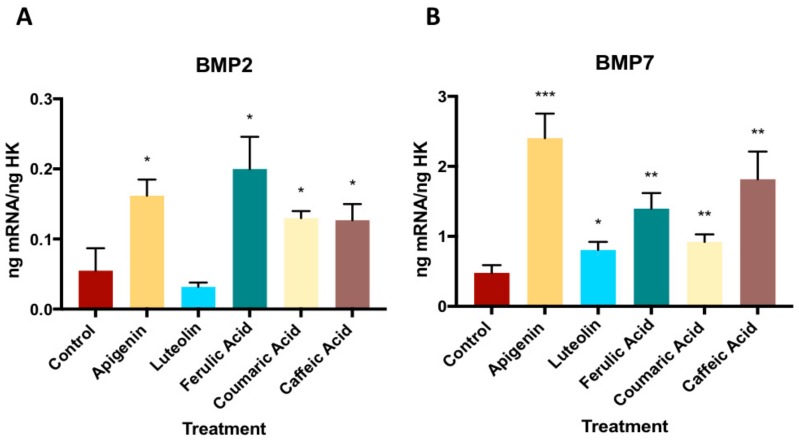
The effect of phenolic compounds on gene expression of BMP2 and BMP7. * *p* < 0.05, ** *p* < 0.005, *** *p* < 0.001. Bone morphogenetic proteins 2 and 7 (BMP-2 and BMP-7).

Figure 2 depicts the effect of the treatment with 10^−6^ M of each phenolic compound on the gene expression of BMP2 and BMP7 by MG63 cells. As observed, the gene expressions of both BMP2 and BMP7 were significantly increased versus the controls by the treatment with each compound, except for the expression of BMP2 by luteolin treatment (See Supplementary Table S1).

### 3.3. Effect of Phenolic Compounds on Gene Expression of OPG- RANKL Complex

**Figure 3:**
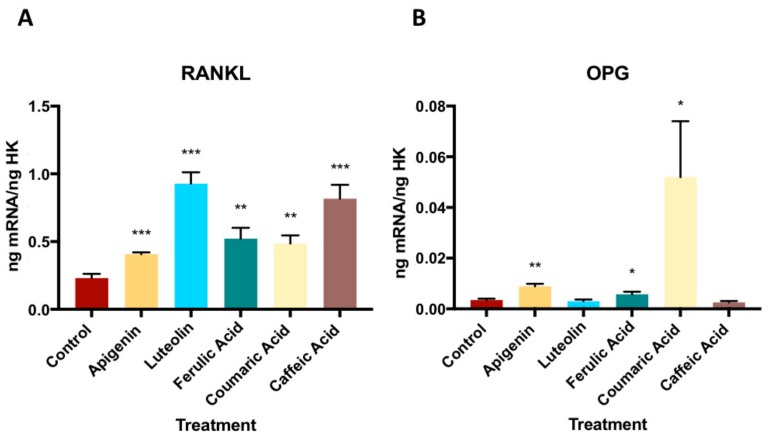
The effect of phenolic compounds on gene expression of OPG-RANKL complex. * *p* < 0.05, ** *p* < 0.005, *** *p* < 0.001. OPG- RANKL: Osteoprotegerin- receptor activator of nuclear factor-kappaB ligand.

As shown in Figure 3, the RANKL expression by the MG63 cells increased after the treatment with 10^−6^ M of each phenolic compound, and their OPG expression was significantly increased by the treatment with apigenin, ferulic acid, or coumaric acid, but not by the treatment with luteolin or caffeic acid. Thus, the OPG-RANKL complex is increased only with apigenin and coumaric acid treatment (See Supplementary Table S1).

### 3.4. Effect of Phenolic Compounds on the Gene Expression of RUNX-2, ALP, COL-I, OSX and OSC

**Figure 4:**
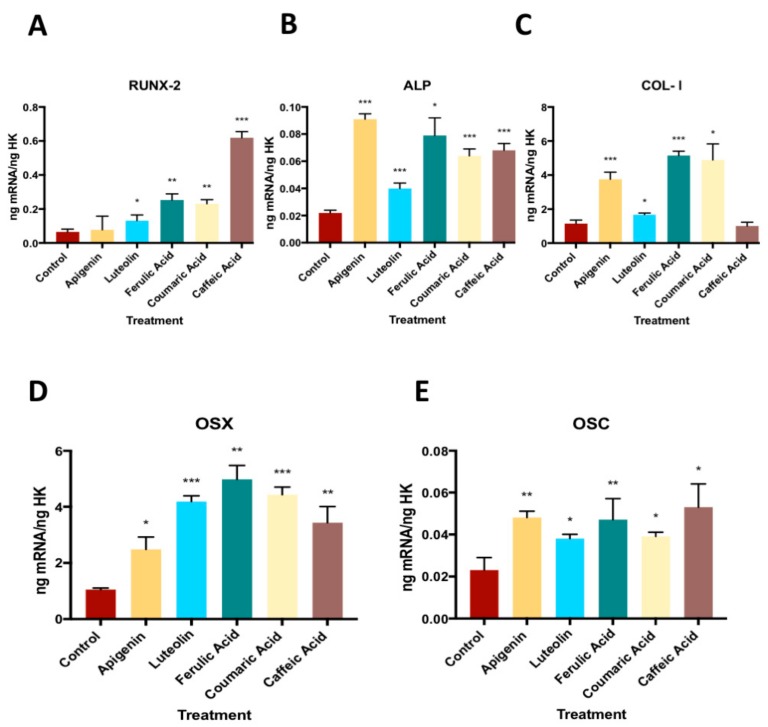
The effect of phenolic compounds on the gene expression of RUNX-2, ALP, COL-I, OSX, and OSC. * *p* < 0.05, ** *p* < 0.005, *** *p* < 0.001. RUNX-2: Run-related transcription factor 2, ALP: Alkaline phosphatase, COl-I: Collagen type I, OSX: Osterix, OSC: Osteocalcin.

Figure 4 displays the q-RT-PCR results for the expression of osteoblast differentiation markers RUNX-2, ALP, COL-I, OSX, and OSC. The treatment with each phenolic compound increased the expression of all markers, except for COL-I, which was not affected by caffeic acid treatment, and RUNX-2, which was not affected by apigenin treatment (See Supplementary Table S1).

## 4. Discussion

This study found that treatment for 24 h with 10^−6^ M of caffeic acid, ferulic acid, coumaric acid, apigenin, or luteolin, phenolic compounds present in EVOO changes the expression of growth and differentiation markers by MG63 osteoblasts. Although others phenolic compounds are present in olive oil, such as oleuropein aglycone, oleacein, oleocanthal, hydroxytyrosol and tyrosol [22], the present study only included those compounds and the dose that had a significant effect on growth, differentiation and the antigenic profiles of the MG63 cell line [6,16,17]. These findings indicate that bone physiology may be modulated by the phenolic compounds in EVOO, supporting previous observations on their action on osteoblasts at different levels, favoring bone tissue regeneration.

The expression of TGFβ1 and its receptors was increased by treatment with each phenolic compound except for coumaric acid, which did not change the expression of TGFβ-R2 or TGFβ-R3, and luteolin, which had no effect on TGFβ-R3 expression. The TGF-β superfamily, which includes TGF-β isoforms 1,2, and 3, BMPs, activins, and nodal proteins, is a set of transmembrane glycoproteins involved in the signaling cascade that regulates osteoblast differentiation and mineralization [23,24,25]. Moderate doses of TGF-β (1ng/mL and 10ng/mL) have been found to improve the proliferation and differentiation of osteoblasts [26,27]. An increased expression of this marker has also been reported in osteoblasts treated with other bone tissue regenerative therapies [23,28,29].

BMPs, of which more than 20 have been identified, stimulate bone tissue regeneration and bone cell differentiation, as demonstrated by their effects on ALP and the expression of COL-I, OSC, and OPN, among other cell differentiation markers [30,31]. In the present study, the expression of both BMP2 and BMP7 was higher after treatment for 24 h with 10^−6^ M of each phenolic compound except for luteolin, which only increased the expression of BMP7. These findings are consistent with the results obtained for phenolic compounds in other vegetables. For instance, flavonoids from natural extracts (e.g., icariin or naringin) were found to stimulate bone tissue regeneration by acting on the BMP signaling pathway, favoring the production of molecules closely related to bone metabolism, including ALP, OSC, and OPN [32,33,34,35]. Likewise, daidzein, a soy phytoestrogen, was reported to stimulate osteoblast proliferation and differentiation by activating the BMP/Smad signaling pathway [36].

The osteogenic differentiation of mesenchymal cells is regulated by various transcriptional factors such as OSX and RUNX-2/Cbfa1, which are essential for the regulation of genes involved in producing bone extracellular matrix proteins (e.g., ALP, COL-I, bone sialoprotein [BSP], OSC, and OPN) and for the induction of bone mineralization [37]. OSX regulates the expression of osteoblast differentiation markers (e.g., RUNX-2 and osteonectin [OSN]) and is indispensable for bone proliferation, differentiation, and formation [38,39]. The expressions of OSX and RUNX-2 significantly increased by the treatment with each EVOO phenolic compound in our study, except for apigenin, which significantly increased the expression of OSX, although to a lesser extent versus the other studied compounds, but had no significant impact on the RUNX-2 expression. These effects were accompanied by an increase in ALP and OSC expression after all the treatments and by an increase in COL-I and OPN expression after almost all of them. The treatment of MC3T3-E1 murine osteoblast cells with ugonin K, a flavonoid present in the herbaceous fern-like plant *Helminthostachys zeylanica*, was reported to regulate the expression of RUNX-2 and OSX and enhance ALP activity, BSP and OSC expression, and mineralization [40]. Likewise, salvianolic acid B, a phenolic acid from the Chinese medicinal plant *Salvia Miltiorrhiza*, was found to increase the expression by mesenchymal cells of ALP, OPN, RUNX-2, and OSC [41]. The increases in the expression of BMP2, COL-I, ALP, and RUNX-2 genes by the cells and in their ALP activity and mineralization were also observed after treatment with extracts of *Davallia formosana*, a petrophylic fern widely used in traditional Chinese medicine [42]. ALP and COL-I are early matrix mineralization markers, whereas OSC and OPN are osteogenic maturation markers [43,44] that indicate the differentiation and maturation of bone. Taken together, these data suggest that polyphenols in EVOO (e.g., caffeic acid, ferulic acid, coumaric acid, apigenin, and luteolin) may have a beneficial effect on bone physiology, favoring osteoblast maturation and differentiation.

The in vitro treatment of MG63 osteoblast cells with various EVOO phenolic compounds was found to induce cell maturation by increasing the synthesis of ALP and reducing the expression of antigens involved in osteoblast immune functions, which may contribute to improving bone mineral density [16]. Similar effects were observed in osteoblast cells after treatment with extracts of different polyphenol-rich EVOO varieties (Picual, Arbequina, Picudo, and Hojiblanca), observing a greater impact for oils with higher content of phenolic compounds [17]. According to these studies, osteoblast maturation and differentiation can be increased in vitro by the treatment either with EVOO extracts or with the phenolic fraction isolated from EVOO.

In contrast, an in vitro study found that treatment of mesenchymal cells with high doses (100 μM) of hydroxytyrosol reduced the expression of osteoblast differentiation markers and inhibited osteoblastogenesis [45], while the same dose of apigenin was reported to inhibit osteoblast differentiation markers (COL-I production, ALP, and calcium deposits) in osteoblasts of murine origin [46]. Previous studies by our group concluded that the dose level is a determinant factor, with high doses having a toxic effect on cells [6].

In the present study, the expression of RANKL was increased by the treatment with all studied polyphenols, especially luteolin, while the expression of OPG increased by apigenin, ferulic acid, and coumaric acid, but not by luteolin or caffeic acid. RANKL belongs to the family of the tumor necrosis factor (TNF) ligand and is expressed on mesenchymal cells, preosteoblasts, and T cells. The binding of RANKL with its receptor (RANK) stimulates osteoclast activation and differentiation [47]. However, OPG is a RANKL receptor that inhibits osteoclast differentiation and function, because its interaction with RANKL can prevent it from binding to RANK [48]. The increased expression of these markers and the higher OPG/RANKL ratio may result from activation of the Wnt canonical signaling pathway, which would favor bone tissue formation [49]. The phenolic compounds from various vegetable species have been found to promote osteoblast differentiation through the expression of these markers, which are closely related to bone maturation [50,51,52,53,54].

Recent animal studies confirmed that various phenolic compounds in different vegetable species (e.g., eugenol or salicylic acid) exert beneficial effects on bone [55,56]. Further, in vivo studies are required to verify our in vitro findings on the effects on bone metabolism of the phenolic compounds in EVOO.

## 5. Conclusions

These findings suggest that EVOO phenolic compounds (e.g., caffeic acid, ferulic acid, coumaric acid, apigenin, and luteolin) may have a beneficial effect on bone physiology, exerting a stimulatory effect on markers involved in osteoblast proliferation, differentiation and maturation. These results support the protective effect of the phenolic compounds in EVOO against bone pathologies, like osteoporosis. However further research is warranted to explore their usefulness in the management of bone diseases.

## Figures and Tables

**Table 1:** Primer sequences for the amplification of cDNA by real-time PCR.

Gene	Sense Primer	Antisense Primer	Amplicon (bp)
TGFβ1	5’-TGAACCGGCCTTTCCTGCTTCTCATG-3´	5´-GCGGAAGTCAATGTACAGCTGCCGC-3´	152
TGFβ- R1	5´-ACTGGCAGCTGTCATTGCTGGACCAG-3´	5´-CTGAGCCAGAACCTGACGTTGTCATATCA-3´	201
TGFβ- R2	5´-GGCTCAACCACCAGGGCATCCAGAT-3´	5´-CTCCCCGAGAGCCTGTCCAGATGCT-3´	139
TGFβ- R3	5´-ACCGTGATGGGCATTGCGTTTGCA-3´	5´-GTGCTCTGCGTGCTGCCGATGCTGT-3´	173
RUNX-2	5´-TGGTTAATCTCCGCAGGTCAC-3´	5´-ACTGTGCTGAAGAGGCTGTTTG-3´	143
OSX	5´-TGCCTAGAAGCCCTGAGAAA-3´	5´-TTTAACTTGGGGCCTTGAGA-3´	205
BMP2	5´-TCGAAATTCCCCGTGACCAG-3´	5´-CCACTTCCACCACGAATCCA-3´	142
BMP7	5´-CTGGTCTTTGTCTGCAGTGG-3´	5´-GTACCCCTCAACAAGGCTTC-3´	202
COL-I	5´-AGAACTGGTACATCAGCAAG-3´	5´-GAGTTTACAGGAAGCAGACA-3´	471
OSC	5´-CCATGAGAGCCCTCACACTCC-3´	5´-GGTCAGCCAACTCGTCACAGTC-3´	258
ALP	5´-CCAACGTGGCTAAGAATGTCATC-3´	5´-TGGGCATTGGTGTTGTACGTC-3´	175
RANKL	5´-ATACCCTGATGAAAGGAGGA-3´	5´-GGGGCTCAATCTATATCTCG-3´	202
OPG	5´-ATGCAACACAGCACAACATA-3´	5´-GTTGCCGTTTTATCCTCTCT-3´	198

Quantitative RT-PCR (q-RT-PCR) was performed using the SsoFast™ EvaGreen® Supermix Kit (Bio-Rad laboratories) in accordance with the manufacturer`s protocol, as described elsewhere [22]. PCR: Polymerase chain reaction.

## Supplementary Materials

The following are available online at https://www.mdpi.com/2072-6643/11/8/1722/s1, Table S1: Data for the gene expression of osteoblast-related markers. Mean, standard deviation and *p* value information after treatment with phenolic compounds vs control.
