# Supplementary material for: Bone Protective Effect of Extra-Virgin Olive Oil Phenolic Compounds by Modulating Osteoblast Gene Expression
Source: Nutrients. 2019 Jul 25;11(8):1722. doi: 10.3390/nu11081722 (PMC6722737; doi:10.3390/nu11081722)
Supplement: Supplementary file 1 [file nutrients-11-01722-s001.pdf]

Table S1: Data for the gene expression of osteoblast-related markers. Mean, standard deviation and p value information after treatment with phenolic compounds *vs* control.

**TGF-β1 Expression**	Mean ± s.d.	P valor	95% Confidence Interval<br>Lower limit	Upper limit
Control	4,09 ± 1,11			
Apigenin 10^{-6}M	8,33 ± 0,38	0,003	-6,13	-2,33
Luteolin 10^{-6}M	13,87 ± 3,11	0,007	-15,08	-4,47
Ferulic acid 10^{-6}M	9,16 ± 0,60	0,002	-7,10	-3,02
Cumaric acid 10^{-6}M	27,31 ± 2,40	0,001	-28,26	-18,16
Cafeic acid 10^{-6}M	14,56 ± 4,00	0,012	-17,12	-3,79

**TGFβ1-R1 Expression**	Mean ± s.d.	P valor	95% Confidence Interval<br>Lower limit	Upper limit
Control	0.561±0.099			
Apigenin 10^{-6}M	1.293±0.202	0.005	-1.094	-0.369
Luteolin 10^{-6}M	1.180±0.053	0.001	-0.799	-0.437
Ferulic acid 10^{-6}M	3.580±0.740	0.002	-4.21	-1.82
Cumaric acid 10^{-6}M	1.787±0.174	0.0001	-1.547	-0.903
Cafeic acid 10^{-6}M	1.903±0.479	0.009	-2.127	-0.556

**TGFβ1-R2 Expression**	Mean ± s.d.	P valor	95% Confidence Interval<br>Lower limit	Upper limit
Control	0.926±0.144			
Apigenin 10^{-6}M	2.098±0.360	0.007	-1.798	-0.545
Luteolin 10^{-6}M	1.066±0.26	0.471	-0.627	0.347
Ferulic acid 10^{-6}M	3.082±0.23	0.0001	-2.603	-1.707
Cumaric acid 10^{-6}M	3.10±0.59	0.004	-3.160	-1.198
Cafeic acid 10^{-6}M	3.150±0.20	0.0001	-2.627	-1.830

**TGFβ1-R3 Expression**	Mean ± s.d.	P valor	95% Confidence Interval<br>Lower limit	Upper limit
Control	0.279±0.003			
Apigenin 10^{-6}M	1.618±0.199	0.007	-1.835	-0.842
Luteolin 10^{-6}M	0.297±0.070	0.674	-0.131	0.094
Ferulic acid 10^{-6}M	0.215±0.01	0.023	0.020	0.107
Cumaric acid 10^{-6}M	0.310±0.150	0.757	-0.414	0.351
Cafeic acid 10^{-6}M	0.738±0.20	0.018	-0.789	-0.129

**BMP2 Expression**	Mean ± s.d.	P valor	95% Confidence Interval Lower limit	Upper limit
Control	0.055±0.032			
Apigenin 10^{-6}M	0.162±0.023	0.013	-0.170	-0.041
Luteolin 10^{-6}M	0.032±0.006	0.304	-0.030	0.076
Ferulic acid 10^{-6}M	0.20±0.046	0.011	-0.236	-0.054
Cumaric acid 10^{-6}M	0.13±0.01	0.023	-0.138	-0.017
Cafeic acid 10^{-6}M	0.127±0.023	0.037	-0.137	-0.006

**BMP7 Expression**	Mean ± s.d.	P valor	95% Confidence Interval Lower limit	Upper limit
Control	0.479±0.11			
Apigenin 10^{-6}M	2.404±0.35	0.001	-2.53	-1.32
Luteolin 10^{-6}M	0.807±0.114	0.025	-0.589	-0.066
Ferulic acid 10^{-6}M	1.397±0.221	0.003	-1.31	-0.516
Cumaric acid 10^{-6}M	0.921±0.108	0.009	-0.696	-0.187
Cafeic acid 10^{-6}M	1.815±0.396	0.005	-1.998	-0.674

**OPG Expression**	Mean ± s.d.	P valor	95% Confidence Interval Lower limit	Upper limit
Control	0.0035±0.0005			
Apigenin 10^{-6}M	0.0089±0.001	0.002	-0.007	-0.0032
Luteolin 10^{-6}M	0.0030±0.0007	0.367	-0.0009	0.0019
Ferulic acid 10^{-6}M	0.0058±0.001	0.027	-0.0041	-0.0004
Cumaric acid 10^{-6}M	0.052±0.022	0.019	-0.084	-0.013
Cafeic acid 10^{-6}M	0.0026±0.0005	0.105	-0.0002	0.0020

**RANKL Expression**	Mean ± s.d.	P valor	95% Confidence Interval Lower limit	Upper limit
Control	0.231±0.031			
Apigenin 10^{-6}M	0.407±0.013	0.001	-0.23	-0.12
Luteolin 10^{-6}M	0.928±0.084	0.0001	-0.58	-0.55
Ferulic acid 10^{-6}M	0.522±0.08	0.005	-0.43	-0.15
Cumaric acid 10^{-6}M	0.485±0.061	0.003	-0.36	-0.14
Cafeic acid 10^{-6}M	0.817±0.103	0.001	-0.76	-0.41

**Runx2 Expression**	Mean ± s.d.	P valor	95% Confidence Interval Lower limit	Upper limit
Control	0.0657±0.016			
Apigenin 10^{-6}M	0.078±0.08	0.08	-0.219	0.193
Luteolin 10^{-6}M	0.132±0.033	0.037	-0.127	-0.006
Ferulic acid 10^{-6}M	0.253±0.036	0.001	-0.252	-0.123
Cumaric acid 10^{-6}M	0.231±0.024	0.001	-0.213	-0.118
Cafeic acid 10^{-6}M	0.619±0.036	0.0001	-0.616	-0.489

ALP Expression	Mean ± s.d.	P valor	95% Confidence Interval Lower limit	Upper limit
Control	0.022±0.002			
Apigenin 10^{-6}M	0.091±0.004	0.0001	-0.068	-0.060
Luteolin 10^{-6}M	0.040±0.004	0.0001	-0.0227	-0.013
Ferulic acid 10^{-6}M	0.079±0.013	0.015	-0.088	-0.026
Cumaric acid 10^{-6}M	0.064±0.005	0.0001	-0.051	-0.031
Cafeic acid 10^{-6}M	0.068±0.005	0.0001	-0.056	-0.036

Col-I Expression	Mean ± s.d.	P valor	95% Confidence Interval Lower limit	Upper limit
Control	1.15±0.21			
Apigenin 10^{-6}M	3.76±0.41	0.001	-3.35	-1.86
Luteolin 10^{-6}M	1.68±0.09	0.035	-0.98	-0.071
Ferulic acid 10^{-6}M	5.15±0.25	0.0001	-4.53	-3.64
Cumaric acid 10^{-6}M	4.89±0.94	0.017	-5.94	-1.52
Cafeic acid 10^{-6}M	1.01±0.22	0.468	-0.35	0.63

OSX Expression	Mean ± s.d.	P valor	95% Confidence Interval Lower limit	Upper limit
Control	1.052±0.056			
Apigenin 10^{-6}M	2.482±0.44	0.03	-2.51	-0.33
Luteolin 10^{-6}M	4.188±0.207	0.0001	-3.48	-2.79
Ferulic acid 10^{-6}M	4.979±0.500	0.005	-5.14	-2.70
Cumaric acid 10^{-6}M	4.434±0.27	0.0001	-3.830	-2.93
Cafeic acid 10^{-6}M	3.431±0.577	0.002	-3.309	-1.45

OSC Expression	Mean ± s.d.	P valor	95% Confidence Interval Lower limit	Upper limit
Control	0.023±0.006			
Apigenin 10^{-6}M	0.048±0.003	0.004	-0.036	-0.013
Luteolin 10^{-6}M	0.038±0.002	0.023	-0.025	-0.003
Ferulic acid 10^{-6}M	0.047±0.01	0.003	-0.033	-0.01
Cumaric acid 10^{-6}M	0.039±0.002	0.014	-0.026	-0.005
Cafeic acid 10^{-6}M	0.053±0.011	0.018	-0.050	-0.005
